# Single Nucleotide Polymorphism Array Lesions, *TET2, DNMT3A*, *ASXL1* and *CBL* Mutations Are Present in Systemic Mastocytosis

**DOI:** 10.1371/journal.pone.0043090

**Published:** 2012-08-15

**Authors:** Fabiola Traina, Valeria Visconte, Anna M. Jankowska, Hideki Makishima, Christine L. O’Keefe, Paul Elson, Yingchun Han, Fred H. Hsieh, Mikkael A. Sekeres, Raghuveer Singh Mali, Matt Kalaycio, Alan E. Lichtin, Anjali S. Advani, Hien K. Duong, Edward Copelan, Reuben Kapur, Sara T. Olalla Saad, Jaroslaw P. Maciejewski, Ramon V. Tiu

**Affiliations:** 1 Department of Translational Hematology and Oncology Research, Taussig Cancer Institute, Cleveland Clinic, Cleveland, Ohio, United States of America; 2 Hematology and Hemotherapy Center, INCT do Sangue, University of Campinas, Campinas, São Paulo, Brazil; 3 Department of Quantitative Health Sciences, Cleveland Clinic, Cleveland, Ohio, United States of America; 4 Department of Pathobiology, Lerner Research Institute and Allergy and Immunology, Respiratory Institute, Cleveland Clinic, Cleveland, Ohio, United States of America; 5 Department of Hematologic Oncology and Blood Disorders, Taussig Cancer Institute, Cleveland Clinic, Cleveland, Ohio, United States of America; 6 Department of Pediatrics, Herman B Wells Center for Pediatric Research, Indiana University of School of Medicine, Indianapolis, Indiana, United States of America; Institut national de la santé et de la recherche médicale (INSERM), France

## Abstract

We hypothesized that analysis of single nucleotide polymorphism arrays (SNP-A) and new molecular defects may provide new insight in the pathogenesis of systemic mastocytosis (SM). SNP-A karyotyping was applied to identify recurrent areas of loss of heterozygosity and bidirectional sequencing was performed to evaluate the mutational status of *TET2, DNMT3A, ASXL1, EZH2, IDH1/IDH2* and the *CBL* gene family. Overall survival (OS) was analyzed using the Kaplan-Meier method. We studied a total of 26 patients with SM. In 67% of SM patients, SNP-A karyotyping showed new chromosomal abnormalities including uniparental disomy of 4q and 2p spanning *TET2*/*KIT* and *DNMT3A.* Mutations in *TET2, DNMT3A, ASXL1* and *CBL* were found in 23%, 12%, 12%, and 4% of SM patients, respectively. No mutations were observed in *EZH2* and *IDH1/IDH2*. Significant differences in OS were observed for SM mutated patients grouped based on the presence of combined *TET2/DNMT3A/ASXL1* mutations independent of *KIT* (*P* = 0.04) and sole *TET2* mutations (*P*<0.001). In conclusion, *TET2, DNMT3A* and *ASXL1* mutations are also present in mastocytosis and these mutations may affect prognosis, as demonstrated by worse OS in mutated patients.

## Introduction

Mastocytosis is a heterogeneous disease characterized by an accumulation of mast cells (MC) in one or more organs [1], [2]. MCs are derived from CD34^+^/KIT^+^ pluripotent hematopoietic cells in the bone marrow [3]. The clinical course of mastocytosis ranges from ‘asymptomatic’ with normal life expectancy to ‘highly aggressive’ [4]. The 2008 World Health Organization (WHO) classification defines 7 disease-variants: cutaneous mastocytosis (CM), indolent systemic mastocytosis (ISM), SM with an associated clonal hematological non-MC-lineage disease (SM-AHNMD), aggressive SM (ASM), MC leukemia (MCL), MC sarcoma (MCS), and extracutaneous mastocytoma. SM is defined by major and minor SM-criteria, requiring at least one major and one minor or at least three minor SM-criteria to make the diagnosis [5]. The natural history of SM varies significantly between patients; patients with indolent forms do extremely well while some aggressive subtypes may rapidly progress to leukemia. The molecular pathogenesis of mastocytosis involves the acquisition of *KIT* mutations, particularly D816V, which is present in many cases and confers resistance to imatinib [6]–[9]. Despite the availability of diagnostic criteria, new predictive and prognostic biomarkers are needed [10]. We hypothesized that analysis of molecular defects in mastocytosis may shed light on the disease pathogenesis and possibly convey prognostic information that may help in the diagnosis and selection of rational therapies.

**Table 1:** Clinical and laboratory characteristics of mastocytosis patients.

	Systemic Mastocytosis			
	ISM	SM-AHNMD	ASM	MCSΔ
Total no. of patients	15	8	2	1
**Age in years, median (range)**	48 (20–79)	76 (12–79)	72 (67–76)	58
**Sex (M/F)**	7/8	6/2	1/1	0/1
**Clinical characteristics, N (%)**				
**Urticaria pigmentosa**	10 (67)	2 (25)	2 (100)	NA
**Cutaneous symptoms** *	10 (67)	2 (25)	2 (100)	NA
**Constitutional symptoms** †	3 (20)	1 (12.5)	1 (50)	NA
**Mediator-related symptoms** ‡	5 (33)	0	1 (50)	NA
**Weight loss** §	1 (7)	2 (25)	1 (50)	NA
**Hepatomegaly** II	1 (7)	3 (38)	1 (50)	NA
**Splenomegaly** II	3 (20)	5 (62.5)	1 (50)	NA
**Lymphadenopathy** ¶	1 (7)	0	0	NA
**Laboratory characteristics, median (range)**				
**Hemoglobin, g/dL**	13.2 (9.6–16.9)∑	9.6 (7.5–12.8)	9.5 (9.5–9.6)	15.9
**White blood cell count, x10^9^/L**	7.6 (4.3–16.4)	18.5 (4.1–53.4)	23.2 (9.8–36.6)	6.9
**Eosinophil count, ×10^9^/L**	0.1 (0.01–0.8)	0.3 (0–5.5)	0.2 (0.1–0.3)	0.2
**Monocytes count, ×10^9^/L**	0. 5 (0.2–1.2)	2.6 (0.53–14)	1 (0.7–1.2)	0.4
**Platelet count, ×10^9^/L**	269 (123–405)	117 (24–514)	272.5 (246–299)	241
**Albumin, g/dL (3.5–5.0)**	4.4 (2.9–5.1)	4 (2.7–4.5)	2.85 (2.1–3.6)	4.7
**Serum alkaline phosphatase, U/L (40–150)**	84.5 (11–132)	153.5 (75–1621)	192 (167–217)	76
**AST, U/L (7–40)**	15.5 (10–37)	20 (11–97)	18.5 (16–21)	27
**Total bilirubin, mg/dL (0–1.5)**	0.5 (0.2–0.9)	0.6 (0.2–1.3)	0.56 (0.5–0.6)	0.2
**LDH, U/L (100–220)**	174 (107–262)	207 (144–657)	403 (NA)	183
**Serum Tryptase, ng/mL (1.9–13.5)**	70.6 (18.3–922)	71.4 (10.7–324)Π	361 (157–565)	9.8

SM, systemic mastocytosis; ISM, indolent SM; SM-AHNMD, SM with associated non-mast cell lineage disease; ASM, aggressive SM; MSC, mast cell sarcoma; M, male; F, female; AST, aspartate aminotransferase; LDH, lactate dehydrogenase. NA, not available.

* Includes pruritis, flushing, urticaria, and angioedema.

† Includes weight loss, fever, chills, and night sweats.

‡ Includes headache, dizziness/lightheadedness, syncope/presyncope, hypotension, anaphylaxis, palpitation/tachycardia, bronchoconstriction/wheezing, and peptic ulcer disease.

§ Weight loss of >10% of normal body weight over a period of 6 months or less.

II Palpable splenomegaly or hepatomegaly.

¶ Lymphadenopathy on palpation or imaging.

Δ The diagnosis of mast cell sarcoma was made based on a right femoral biopsy (patient 16).

∑ One patient with ISM had anemia at the time of sampling; the causes of anemia were bacterial endocarditis and renal insufficiency related to a proliferative glomerulonephritis (patient 12 of Table 2).

Π One patient who fulfilled criteria for SM-AHNMD had a low tryptase level of 10.7 ng/mL which was taken at the time of AML remission (patient 17 of Table 2).

In this study, we performed single nucleotide polymorphism array (SNP-A) karyotyping analysis in SM patients to define minimally affected genomic regions and identify new mutations in this disease. We also searched for *TET2*, *DNMT3A*, *ASXL1*, *EZH2*, *IDH1/2* and *CBL* gene families mutations, given their potential clinical importance in diseases closely associated with SM like primary myelofibrosis, chronic myelomonocytic leukemia (CMML) and others [11]–[17]. Ultimately, we correlated any lesions present with clinical phenotypes and survival outcomes.

## Methods

### Patients

Ethics statement: The use of human samples for this study was approved by institutional review board (IRB) of the Cleveland Clinic and written informed consent for sample collection was obtained in accordance with the Declaration of Helsinki.

**Figure 1:**
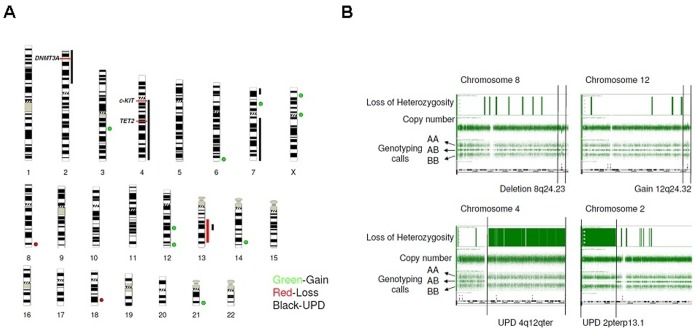
Single nucleotide polymorphism array-based karyotyping (SNP-A) of mastocytosis patients. (A) Overview of all genetic aberrations found by SNP-A analysis in patients with systemic mastocytosis. Green represents gain, red represents loss, black represents somatic uniparental disomy (UPD). UPD involving the *KIT* and *TET2* genes on chromosome 4q and UPD involving the *DNMT3A* gene on chromosome 2p were noted in one patient each, as indicated. (B) Representative SNP-A analysis of loss of heterozygosity (LOH), UPD, and gain by Genotyping Console v3.0. The top track of each panel shows LOH. The second track shows raw copy number for each SNP along the chromosome, while the third track shows allele calls (AA, AB, BB). Each region of genomic change is indicated by vertical black bars.

We studied a total of 26 patients with SM (10 bone marrow aspirates and 16 peripheral blood samples); 15 ISM, 8 SM-AHNMD (5 CMML, 1 acute myeloid leukemia [AML], 1 non-Hodgkin’s lymphoma, and 1 CMML/chronic lymphocytic leukemia), 2 ASM and 1 MCS. The median age at sample collection was 63 years (range 13–77). The median time from diagnosis to sample collection was 23 months (range 0–521). Samples were collected at the Cleveland Clinic between 2003 and 2009. Diagnosis was assigned according to 2008 WHO classification criteria [5]. The clinical and hematologic characteristics of patients are summarized in Table 1. Karyotypic abnormalities detected by metaphase cytogenetics were found in 2/16 SM patients (13%); one patient had trisomy 8 and one patient had an inversion on chromosome 20.

**Table 2:** Mutational status in patients with systemic mastocytosis.

Disease	Sample	Patient	*KIT*	*TET2*	*DNMT3A*	*ASXL1*	*IDH1/2*	*EZH2*	*CBL*
**ISM** ‡ II	**PB**	**1**	wt	wt	wt	wt	wt	wt	wt
	**PB**	**2**	wt	wt	wt	mutant	wt	wt	wt
	**PB**	**3**	mutant	wt	wt	wt	wt	wt	wt
	**PB**	**4**	wt	wt	wt	wt	wt	wt	wt
	**PB**	**5**	wt	wt	wt	wt	wt	wt	wt
	**BM**	**6**	wt	wt	mutant	wt	wt	wt	wt
	**PB**	**7**	mutant	mutant	mutant	wt	wt	wt	wt
	**PB**	**8**	wt	wt	wt	wt	wt	wt	wt
	**BM**	**9**	mutant	wt	wt	wt	wt	wt	wt
	**BM**	**10**	wt	wt	wt	wt	wt	wt	wt
	**BM**	**11**	wt	wt	wt	wt	wt	wt	wt
	**PB**	**12**	wt	wt	wt	wt	wt	wt	wt
	**PB**	**13**	wt	wt	wt	wt	wt	wt	wt
	**PB**	**14**	mutant	wt	wt	wt	wt	wt	wt
	**PB**	**15**	wt	wt	wt	wt	wt	wt	wt
**SM-AHNMD** §	**PB**	**16** *	wt	mutant	mutant	wt	wt	wt	wt
	**PB**	**17**	wt	mutant	wt	wt	wt	wt	mutant
	**BM**	**18**	mutant	mutant	wt	mutant	wt	wt	wt
	**BM**	**19**	mutant	mutant	wt	mutant	wt	wt	wt
	**BM**	**20**	wt	wt	wt	wt	wt	wt	wt
	**BM**	**21**	wt	wt	wt	wt	wt	wt	wt
	**PB**	**22**	mutant	wt	wt	wt	wt	wt	wt
	**PB**	**23** †	mutant	mutant	wt	wt	wt	wt	wt
**Aggressive Mastocytosis**	**BM**	**24**	mutant	wt	wt	wt	wt	wt	wt
	**PB**	**25**	mutant	wt	wt	wt	wt	wt	wt
**Mast Cell Sarcoma**	**BM**	**26**	wt	wt	wt	wt	wt	wt	wt
		**Total**	**10**	**6**	**3**	**3**	**0**	**0**	**1**

ISM, indolent systemic mastocytosis; SM-AHNMD, Systemic mastocytosis with associated clonal hematological non-mast cell lineage disease; wt, wild-type.

* UPD2pterp13.1 (homozygous *DNMT3A* mutation).

† UPD4q12qter (homozygous *KIT* and *TET2* mutation).

‡ Patients with bone marrow mastocytosis: 1, 2, 4, 5, 6, 7, 8, 9, 10, 13, 14, 15; patients with smouldering systemic mastocytosis: 3, 11, 12.

§ Associated hematological non mast cell disease: chronic myelomonocytic leukemia for patients 16, 17, 18, 19 and 22; non-Hodgkin’s lymphoma for patient 20, acute myelogenous leukemia for patient 21, chronic myelomonocytic leukemia and chronic lymphocytic leukemia for patient 23^.^

II Patients with urticaria pigmentosa: 1, 2, 4,6, 7, 9, 10, 14, 15.

**Table 3:** Characteristics of patients carrying *TET2*, *DNMT3A*, *ASXL1* and *CBL* family mutation.

Patient	WHO dx	Sex	Age,y	Cytogenetics	*KIT D*816V	New mutations			
						Gene	Exon	Nucleotide change	Amino acid change
2	ISM	M	62	NA	Neg.	*ASXL1*	12	c.3658A>T	I1220F
6	ISM	F	77	NA	Neg.	*DNMT3A*	3	c.89A>C	E30A
7	ISM	M	72	NA	Pos.	*TET2*	3	c.1226_1229delCTCC	P409fsX17
						*TET2*	8	c.4011T>A	Y1337X
						*DNMT3A*	23	c.2645G>A	R882H §
16	SM-AHNMD^II^	F	75	46,XX [20]	Neg.	*TET2*	3	c.3058C>T	Q1020X
						*DNMT3A*	19	c.2312G>A	R771Q * §
17	SM-AHNMD^II^	M	75	46,XY [20]	Neg.	*TET2*	3	c.1955_1955delA	Q652fsX48 §
						*CBL*	8	c.1101_1102insCAA	Ins368Q
18	SM-AHNMD^II^	F	72	46,XX [20]	Pos.	*TET2*	11	c.5618T>C	I1873T ‡ §
						*ASXL1*	12	c.2757_2758insA	P920fsX4 ‡
19	SM-AHNMD^II^	M	74	46,XY,?inv(20)(q11.2q13) [20]	Pos.	*TET2*	11	c.5711A>G	H1904R §
						*ASXL1*	12	c.1772_1773insA	Y591X §
23	SM-AHNMD¶	M	75	46,XY,add(8)(q24) [20]	Pos.	*TET2*	3	c.4delG	E2fsX13 †

WHO, World Health Organization; SM, systemic mastocytosis; ISM, indolent SM; SM-AHNMD, SM with associated non-mast cell lineage disease; ASM, aggressive SM; MSC, mast cell sarcoma; M, male; F, female; dx, diagnosis; Age, y, years; Neg., negative; Pos., positive.

* UPD2pterp13.1 (homozygous mutation).

† UPD4q12qter (homozygous mutation).

‡ Germ-line confirmation.

§ Mutations reported at http://www.sanger.ac.uk.

II Associated hematological non mast cell disease: chronic myelomonocytic leukemia.

¶ Associated hematological non mast cell disease: chronic myelomonocytic leukemia and chronic lymphocytic leukemia.

**Figure 2:**
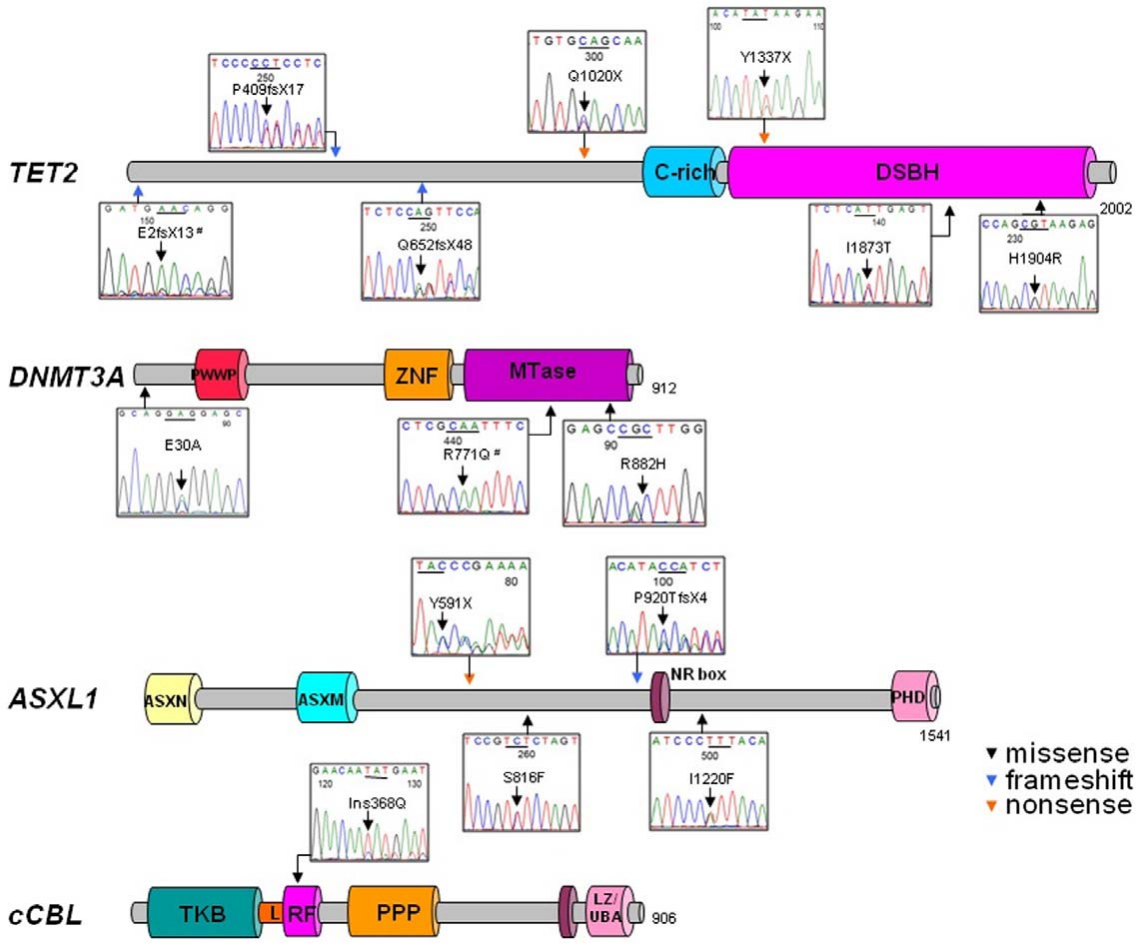
Localization of mutations identified in systemic mastocytosis. In a cohort of 26 patients with systemic mastocytosis, 14 mutations were identified. Genomic sequencing of protein-coding regions and splice sites revealed missense (black), nonsense (orange), and frameshift mutations (blue) in *TET2*, *DNMT3A*, *ASXL1,* and *CBL*. Most mutations were found in conserved domains and specific known conserved motifs and domains are shown for each protein: cysteine-rich region (C-rich-), double strand â helix (DSBH), PWWP domain (characterized by the presence of a highly conserved proline–tryptophan–tryptophan–proline motif), ADD (*ATRX, DNMT3,* and *DNMT3L*)-type zinc finger (ZNF) domain, methyltransferase (MTase) domain, amino-terminal ASX homology (ASXN) region, ASXM domain, nuclear receptor coregulator binding (NR box) motifs, carboxyterminal plant homeodomain (PHD) domain, tyrosine kinase binding (TKB) domain, linker sequence (L), RF domain (RF), proline-rich region (PPP), and leucine zipper LZ/ubiquitin-associated domain (UBA). Two changes occurred in a homozygous state, as indicated by the symbol # and the others in heterozygous state.

### Single Nucleotide Polymorphism Array (SNP-A) Analysis

Mononuclear cells (MNCs) from bone marrow or peripheral blood samples were separated on Ficoll Hypaque density gradients (1.077) at 400 g for 30 min. Genomic DNA from MNCs cells was extracted using Gentra Puregene DNA Extraction kit (Gentra Systems, Inc., MN) according to the manufacturer’s instructions. The Affymetrix GeneChip Human Mapping 250 K Array and Genome-Wide Human SNP Array 6.0 (Affymetrix, Santa Clara, CA) were used for SNP-A analysis of genomic DNA as previously described [18], [19]. Germ-line encoded copy number variants (CNVs) and non-clonal areas of uniparental disomy (UPD) were excluded from further analysis by utilizing a bioanalytic algorithm which was based on the results of SNP-A karyotyping [11], [13] in an internal control series (n = 1003) and reported in the Database of Genomic Variants (http://projects.tcag.ca/variation/; accessed February 4, 2009.

**Table 4:** Clinical and laboratory features of mastocytosis patients stratified according to *TET2* mutations.

	*TET2* wt	*TET2* mutant	*P* value
Total no. of patients	20	6	
**Age in years, median (range)**	54 (12–79)	76 (73–77)	**0.01**
**Sex (M/F)**	10/10	2/4	0.65
**Clinical characteristics, N (%)**			
**Urticaria pigmentosa**	11 (55)	3 (52)	1.0
**Cutaneous symptoms** *	12 (60)	2 (33)	0.37
**Constitutional symptoms** †	4 (20)	1 (17)	1.0
**Mediator-related symptoms** ‡	6 (30)	0	0.28
**Weight loss** §	2 (10)	2 (33)	0.22
**Hepatomegaly** II	2 (10)	3 (50)	0.06
**Splenomegaly** II	5 (25)	4 (67)	0.14
**Lymphadenopathy** ¶	1 (5)	0	1.0
**Laboratory characteristics, median (range)**			
**Hemoglobin, g/dL**	12.6 (7.5–16.9)	9.7 (8.6–13.0)	0.08
**White blood cell count, ×10^9^/L**	8.2 (4.1–36.6)	26.0 (4.3–53.4)	0.32
**Eosinophil count, ×10^9^/L**	0.2 (0.02–5.5)	0.3 (0–0.8)	0.95
**Monocytes count, ×10^9^/L**	0.5(0.2–4.9)	2.6(0.42–14.0)	**0.009**
**Platelet count, ×10^9^/L**	266(111–514)	110(24–329)	**0.009**
**Albumin, g/dL (3.5–5.0)**	4.3(2.1–5.1)	4.1(3.7–4.5)	0.59
**Serum alkaline phosphatase, U/L (40–150)**	96(11–217)	154(75–1621)	0.09
**AST, U/L (7–40)**	16(10–97)	22(11–61)	0.23
**Total bilirubin, mg/dL (0–1.5)**	0.5(0.2–.9)	0.8(0.5–1.3)	0.08
**LDH, U/L (100–220)**	174(107–403)	207(176–657)	0.14
**Serum Tryptase, ng/mL (1.9–13.5)**	71.0(9.8–922.0)	129.0(31.0–324.0)	0.79

Symbols and Abbreviations: AST, aspartate aminotransferase, LDH, Lactate dehydrogenase, wt, wild-type; NA, not available.

* Includes pruritus, flushing, urticaria, and angioedema.

† Includes weight loss, fever, chills, and night sweats.

‡ Includes headache, dizziness/lightheadedness, syncope/presyncope, hypotension, anaphylaxis, palpitation/tachycardia, bronchoconstriction/wheezing, and peptic ulcer disease.

§ Weight loss of >10% of normal body weight over a period of 6 months or less.

II Palpable splenomegaly or hepatomegaly.

¶ Lymphadenopathy on palpation or imaging.

### Mutational Analysis of Patients with Mastocytosis

We sequenced *KIT, TET2*, *DNMT3A*, *ASXL1*, *EZH2*, *IDH* and *CBL* gene families in all 26 patients. Direct genomic sequencing was performed on coding exons, for *KIT* (exon 17), *TET2* (all exons), *DNMT3A* (all exons), *ASXL1* (exon 12), *EZH2* (all exons), *IDH1* (exon 4), *IDH2* (exon 4), *CBL* (exons 8–9), *CBLB* (exons 9–10), and *CBLC* (exons 7–8) as previously described [11]–[14], [20], [21]. Primer sequences and conditions used are described in *Table S1*. For germ-line confirmation, mutations were analyzed in non-clonal CD3^+^ cells when DNA was available. Bidirectional sequencing was performed by standard techniques using an ABI 3730×l DNA analyzer (Applied Biosystems, Foster City, CA). All mutations were scored as pathogenic on the basis of the observation that they were not detected in normal samples and were not found in published SNP databases (dbSNP, http://www.ncbi.nlm.nih.gov/projects/SNP) and/or they were not reported as SNPs in previous publications.

**Figure 3:**
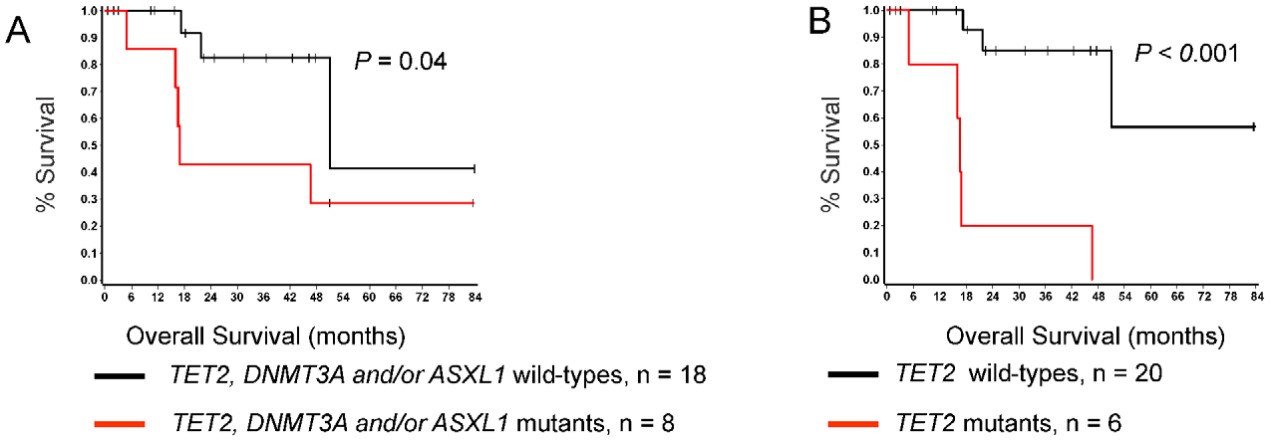
Kaplan-Meier survival curves estimated according to presence of specific mutations or accumulation of several mutations in patients with systemic mastocytosis. Differences in OS for SM patients are shown (A-B). For each group number of analyzed cases and *P* value are presented, respectively.

### Statistical Analysis

Fisher’s exact test for the analysis of categorical data and the exact Wilcoxon rank sum test were used for measured data. Overall survival (OS) was measured from the day of sampling to last follow up or death from any cause (patients lost to follow-up were censored) and was summarized using the Kaplan-Meier method. Univariable analyses were conducted using exact logrank test and Tarone-Ware trend tests. Multivariable analyses were not performed due to the small number of patient deaths. Results were analyzed for data collected as of January 2011. All *p* values were two sided and *p* values ≤.05 indicated statistical significance. Statistical analyses were performed using SAS version 9.1 (SAS Inc., Cary, NC) and StatXact-9 (Cytel Inc., Cambridge, MA).

## Results

### SNP-A-based Detection of Karyotypic Abnormalities in Systemic Mastocytosis

SNP-A karyotyping allows for the identification of not only submicroscopic copy number changes but also somatic UPD, not amenable to detection using routine metaphase cytogenetics. SNP-A-based karyotyping was performed on a subset of patients with SM (n = 18; 7 bone marrow aspirates and 11 peripheral blood samples). For the purpose of this study, we only included lesions which did not overlap with CNVs and germ- line regions of homozygosity present in an internal control cohort and external databases (see Methods). SNP-A analysis identified a total of 22 new lesions (14 gains, 3 losses, and 5 UPD) in 12 patients (5 ISM, 5 SM-AHNMD, 1 ASM and 1 MCS). The frequency of SNP-A lesions was 57% (4/7) in bone marrow and 72% (8/11) in peripheral blood samples. The most frequently affected chromosomes were 2, 7, 12, 13, 14 and X. Somatic UPD was only found in SM-AHNMD and ASM and it involved chromosomes 2p, 4q, 7p and 13q. UPD4q spanning *KIT* (4q12) and *TET2* (4q24), and UPD2p spanning *DNMT3A* (2p23), were observed in one case each (Fig. 1A, B) (Table 1). Based on the paradigm that areas of somatic UPD contain homozygous mutations, we sequenced *TET2* and *DNMT3A.* We also searched for mutations in other genes known to be involved in myeloid diseases that share pathophysiologic, morphologic, and clinical similarities with mastocytosis, such as CMML and myelofibrosis [15], [22].

### Mutations in TET2, DNMT3A, ASXL1, EZH2, IDH and CBL Families in Systemic Mastocytosis

We sequenced *TET2, DNMT3A*, *ASXL1*, *EZH2* and the *IDH1/2* and *CBL* gene families in patients with SM identifying 14 mutations in 8/26 (31%) patients. By sample source, mutations involving these genes were found in 31% (5/16) in peripheral blood and 30% (3/10) in bone marrow samples. A total of 7 *TET2* mutations were found in 6/26 (23%) patients, including one patients with 2 mutations (3 frameshift, 2 nonsense, and 2 missense). *TET2* mutational frequencies for ISM and SM-AHNMD were 7% (1/15), and 62% (5/8), respectively. The majority of *TET2* mutations were heterozygous, except for one homozygous mutation that was found in a patient with UPD4q. Only one patient (# 7) with ISM was found to be mutated for *TET2*. This patient had 3 minor criteria for SM: presence of *KIT* mutation, bone marrow with mast cells positive for CD2 and CD25 (less than 30% of mast cells in bone marrow) and persistently elevated serum tryptase levels (31 ng/mL). The bone marrow of this patient did not demonstrate any dysplastic changes nor increased bone marrow blasts to suggest an underlying myeloid neoplasm like CMML or MDS.

*DNMT3A* mutations were found in 3/26 (12%) patients, 2/15 ISM (13%), and 1/8 (12.5%) SM-AHNMD. All *DNMT3A* were missense mutations, including two heterozygous and one homozygous mutation, which were found in a SM-AHNMD patient with UPD2p. We also detected *ASXL1* heterozygous mutations (1 frameshift, 1 nonsense, and 1 missense) in 3/26 (12%) patients with SM. *ASXL1* mutations were found in 1/15 ISM and 2/8 SM-AHNMD. Moreover, the controversial *ASXL1* variant, c.1934dupG p.Gly646TrpfsX12, was found in two patients with SM-AHNMD (CMML) which also had other mutations (patient 22 and 23). This variant was not considered a mutation in our cohort, as it has been recently reported not to be a somatic mutation but rather an artifact [23]. A heterozygous *CBL* mutation was found in one patient with SM-AHNMD. Among the patients with SM-AHNMD, all mutated patients had CMML as the associated non-mast cell disease. Of note, *KIT* sequencing showed D816V in 38% of SM patients (ISM, 27%; SM-AHNMD, 50%; ASM, 100%), including one homozygous mutation in a patient with UPD4q. The frequency of *KIT* mutations were 37% (6/16) in peripheral blood and 60% (6/10) in bone marrow samples. No mutations were found in *EZH2, IDH1*/*2, CBLB, and CBLC*. Interestingly, 6 patients, 5/8 (62%) of patients with SM-AHNMD and 1/15 (7%) of patients with ISM, were found to have >1 mutation: *KIT* and *TET2* in 1, *KIT/TET2/DNMT3A* in 1, *KIT*/*TET2*/*ASXL1* in 2, *TET2*/*DNMT3A* in 1, and *TET2*/*CBL* in 1 patient (mutant cases and the corresponding clinical and molecular features are presented in Table 2, 3). A graphical overview of the mutations in affected genes is shown in Fig. 2A.

### Clinical Impact of Mutations Found in Systemic Mastocytosis

Among the new molecular markers studied, *TET2* were the most commonly mutated gene. The prognostic significance of *KIT* mutations has been previously reported [24]. However, the effects of *TET2* and other novel mutations on survival have not been established in SM. Although the number of patients was small, patients with SM-AHNMD showed *TET2* mutations more frequently than patients with other subtypes (63% [5/8] *vs*. 0–7%, *P* = 0.02). In general, those with *TET2* mutations tended to be older (median age 76 *vs. 54*, *P* = 0.01), had higher absolute monocyte counts (median 2.62 *vs.* 0.53, *P* = 0.009) and lower platelets counts (median 110 *vs.* 266, *P* = 0.009) compared to wild type patients (Table 4).

Among patients with SM, 8 died at a median of 17.3 months (range 4.9 - 51.0 months) from the time of sample collection (ISM, n = 1; SM-AHNMD, n = 5; ASM, n = 2). Median follow-up for the 18 patients still alive is 23.6 months (range 0.6 - 89.1 months). Overall, 1- and 2-year survival was estimated to be 95%±4% and 69%±11%, respectively. SM patients with cytogenetic abnormalities detected by SNP-A karyotyping showed no difference in OS (data not shown). However, significant differences in OS were observed when patients were grouped based on the presence of mutations. Patients with *TET2*, *DNMT3A* and/or *ASXL1* mutations independent of *KIT* status, had a worse OS than those with wild-type genes (*P* = 0.04; Fig. 3A). Similarly, *TET2* mutations appeared to confer a poor prognosis (*P<*0.001; Fig. 3B).

## Discussion

*TET2* mutations are the most recent genetic lesions described in mastocytosis. Tefferi *et al* reported a screening of *TET2* mutations in 42 cases, finding the lesion in 29% of cases [25]. In addition to *TET2* sequencing, we applied whole genome scanning technologies in our mastocytosis cohort in order to interrogate the genome for the presence of new genetic alterations in this disease. Although the small sample size and the random nature of the SNP-A defects in our cohort did not allow for more definitive survival analysis, we were able to detect new karyotypic defects in mastocytosis cases, including regions of UPD. The identification of UPD2p in a patient with SM-AHNMD (CMML) indicated the occurrence of *DNMT3A* mutations in mastocytosis, which was confirmed by the detection of a homozygous mutation in this patient and two heterozygous mutations in other 2 patients with ISM. Although one patient (patient 22) with SM-AHNMD had UPD7q flanking the region of *EZH2*, no mutations in *EZH2* were found in the cohort of mastocytosis patients. It is possible that mutations in *EZH2* will be found if a larger number of SM patients would be screened.

*IDH* family mutations confer an enzymatic gain of function that increases 2-hydroxyglutarate (2HG) and consequently heterozygous acquisition of these mutations may be sufficient to facilitate malignant progression [26]–[28]. No *IDH* mutations were found supporting previous findings that *TET2* and *IDH* are mutually exclusive [27]. *ASXL1* gene is involved in the regulation of histone methylation by cooperation with heterochromatin protein-1 to modulate the activity of LSD1 [29], [30] and *ASXL1* mutation was found in three patient with mastocytosis. The identification of *TET2, DNMT3A* and *ASXL1* mutations in mastocytosis suggest that these defects may alter the epigenetic machinery of the hematopoietic cells in myeloid malignancies, including mastocytosis. Interestingly, a *CBL* mutation was found in only one patient with SM-AHNMD (CMML) and it occurred in conjunction with a *TET2* mutation.

Mutations involving genes like *TET2*, *DNMT3A*, *ASXL1*, *EZH2*, *IDH1/2* and *CBL* are found in typical CMML cases not associated with mastocytosis. When CMML with SM cases were compared against CMML without SM, a higher frequency of *TET2* mutations was noted in CMML with SM patients (83% vs 35–49% [11], [31]). The frequencies of *ASXL1* and *CBL* mutations were very similar between CMML with and without SM [31], [32]. Common clinical features observed among SM patients with *TET2* mutations included older age, high absolute monocyte counts, and low platelet counts. More importantly, SM patients with *TET2* mutations showed worse OS as compared with wild type patients. The significant impact of *TET2, DNMT3A*, and *ASXL1* mutations was also statistically significant when comparing combined new molecular markers. The survival differences we found in our study, although based on a limited sample size, suggest the potential prognostic importance of these mutations in this disease. However, this will need to be further confirmed in a larger patient population. Future studies that will include a larger cohort of patients with sorted cell populations will be ideal.

Most of the mutated patients included in this current study are deceased which represents a technical limitation of this study in isolating specific cell subtypes. We successfully sorted mast cells and monocytes from a new patient with ISM and urticaria pigmentosa, and a *TET2* mutation (Q962X) was identified in peripheral blood MNCs, sorted monocytes and sorted mast cells, but not in CD3^+^ cells (data not shown). All together, these data support the hypothesis suggested by Yavuz et al [7], that mastocytosis is a clonal disorder of a pluripotential hematopoietic progenitor cell that gives rise to mast cell and non-mast cell lineages with variable expansion in the peripheral blood of patients with SM.

The identification of *KIT* mutations in MC diseases is important because it confers resistance to protein kinase inhibitors such as imatinib [8].The frequency of the D816V *KIT* mutation in SM is highly variable in the literature, from 29% to virtually all cases [6], [7], [9], [25], [33], and 38% in our SM cohort. Such variability could be due to patient selection, to the sensitivity of the methods used and/or to sample source. Direct DNA sequencing has limited sensitivity in the detection of *KIT* mutations. Similarly, more sensitive techniques, including RT-PCR plus RFLP, PNA-mediated PCR or allele-specific PCR, when used in unmanipulated or enriched samples only produced sensitivities of ∼ 70% [2], [7]. Bone marrow cells and highly enriched (sorted or micromanipulated) MC are recommended [6], [7], [9], but enrichment is not standard in clinical practice. Interesting, in our cohort, not only *KIT* mutations were detected in peripheral blood samples, but also other molecular markers were identified by SNP-A. Detection of *KIT* mutation in peripheral blood of SM patients has already been reported by other authors [6], [7], [34].

In conclusion, our findings support the feasibility of SNP-A analysis in mastocytosis and an increasing possibility that mutations in *TET2*, *DNMT3A*, and *ASXL1* represent a new class of molecular lesions conveying a clonal epigenetic instability phenotype that participates in the pathogenesis of mastocytosis. We also suggest that combined mutations and sole *TET2* mutations are associated with poor OS in SM. We performed a comprehensive analysis of new molecular markers in mastocytosis and found several distinct clinical and biological characteristics of this disease entity associated with specific mutational events. Further investigations are needed to study the mechanistic significance of these mutations and their impact in diagnostic and therapeutic tools in mastocytosis. The frequent occurrence of these genetic mutations in mastocytosis may also allow for their inclusion in the list of clonal markers that may aid in the pathomorphologic classification of mastocytosis just like *KIT* mutations.

## Supporting Information

Table S1 **Primers` sequences and conditions.**
(DOC)
