# Supplementary material for: Single Nucleotide Polymorphism Array Lesions, TET2, DNMT3A, ASXL1 and CBL Mutations Are Present in Systemic Mastocytosis
Source: PLoS One. 2012 Aug 15;7(8):e43090. doi: 10.1371/journal.pone.0043090 (PMC3419680; doi:10.1371/journal.pone.0043090)
Supplement: Table S1 — Primers` sequences and conditions. (DOC) [file pone.0043090.s001.doc]

**Table S1. Primers` sequences and conditions**

| **Gene** | **Exons** | **Forward Primer 5`-3`** | **Reverse Primer 5`-3`** | **Annealing Temperature** | **% of DMSO** |
| --- | --- | --- | --- | --- | --- |
| ***KIT*** | 17 | CCTCCTTACTCATGGTCGGATC | CTGTTTCCTTCACATGCCCC | 55 |  |
| ***TET2*** | 3 (1) | CAGTTTGCTATGTCTAGGTATTCCG | CGGAGACATTTGGTTGACTG | 53 |  |
|  | 3 (2) | TGGAGGAATAAAACGCACAG | TTAGTAGCCTGACTGTTAATGGC | 53 |  |
|  | 3 (3) | TGGTGCTACAGTTTCTGCCTC | AAGCACCATTCATTTCATTTTG | 53 |  |
|  | 3 (4) | TGCTAAATACCTGTTCCTTTCAG | CAGGGACAATGACTGTTCCA | 53 |  |
|  | 3 (5) | CCCCAACCAAAGTAACACAAC | TCCAGTGTATTGTTTGGAGGTC | 53 |  |
|  | 3 (6) | CCCCAACACAGCACTATCTG | TGAGGCTTATGTTGCAAAAGG | 53 |  |
|  | 3 (7) | TCACACCAGGTGCACTTCTC | TTTTGGGTCTTGTTTCCTGC | 53 |  |
|  | 3 (8) | TTGGCCAGACTAAAGTGGAAG | GTGTTTGCTGCTGTTCTTGC | 53 |  |
|  | 3 (9) | AAGATATGTCTGGTCAACAAGCTG | TTCTGCAGCAGTGGTTTGTC | 53 |  |
|  | 3 (10) | GCATCATTGAGACCATGGAG | TCACAAGACACAAGCATCGG | 53 |  |
|  | 4 | GCCCTTAATGTGTAGTTGGGG | TGCTTTGTGTGTGAAGGCTG | 53 |  |
|  | 5 | TGCCTCTTGAATTCATTTGC | GGGTAACCCAATTCTCAGGG | 53 |  |
|  | 6 | TGCAAGTGACCCTTGTTTTG | TTTTATAAATGTAAAAGTGCACGC | 53 |  |
|  | 7 | CAGCTGCACAGCCTATATAATG | TCACTTCATCTAAGCTAATGAATTCTC | 53 |  |
|  | 8 | GGGATTCAAAATGTAAGGGG | TGCAGTGGTTTCAACAATTAAG | 53 |  |
|  | 9 | TGTCATTCCATTTTGTTTCTGG | TCTGCTCCTCAACATGAGATG | 53 |  |
|  | 10 | ACACACACGTTTTCTTTGGG | CAGAACTTACAAGTTGATGGGG | 53 |  |
|  | 11 (1) | TGATCAGAACCCTGACTTTGC | AACTGCTGAAACCATCTCCC | 55 |  |
|  | 11 (2) | CCAGTCTCAGCCGATGG | ATTGACCCATGAGTTGGAGC | 55 |  |
|  | 11 (3) | TGTGTCCAAGGAGGCTTACAC | CTGACAGGTTGGTTGTGGTC | 55 |  |
| ***DNMT3A*** | 2 | TCTCGCCTCCAAAGACCACGATAA | AGTGATGCGGTCATGCACTCAGTA | 62 | 3 |
|  | 3 | ACAGGCCTGGAATGCTACAC | GGCACGTGTGTGTTGTGTG | 62 |  |
|  | 4 | AGCGGTCAATGATCCAAAAC | CTCTCAGGGTATGCTGGTGG | 60 |  |
|  | 5 | GCCACTCCAGTAATTCTGCC | TCACACACACTCGCCACC | 60 |  |
|  | 6 | CATTGTGTTTGAGGCGAGTG | CTTCTGGTTTTCCAGTTCTGC | 56 | 6 |
|  | 7 | GTCCCCTTGAGTGTCAGGTG | GAGGAGCTGGCAGTGGAAG | 60 |  |
|  | 8 | GGGATCAGGGTGGCAGG | AGGCCCTGGGATCAAGAAC | 60 |  |
|  | 9 | AGTTGCAAGGCATGGGG | ACTTCCAGGCCTCCTAGTGC | 60 |  |
|  | 10 | CTCAGAGTCTGGCCTTGAGC | CTGGTGTGGATCTGCCTG | 60 |  |
|  | 11-12 | AGGGGCTGGAGTTTCCTG | AACCTTCCTAAGTGCCTCTGC | 60 |  |
|  | 13 | GAGGGAGAGGCCCTTCG | TGGACACAGTCAGCCAGAAG | 60 |  |
|  | 14-15 | AGGTGTGGAGCCTCCCTTC | CCCACAACCAAGGCTCAG | 60 |  |
|  | 16 | GTGGGAGCTTGGGACACC | TTCGTTTTGCCAGAGTTGC | 60 |  |
|  | 17 | CTCACCTGCCGAGACCAG | CCTCCAGGTGCTGAGTGTG | 62 |  |
|  | 18-19 | CTGTCTGCCTCTGTCCCTG | GGATGAAGCAGCAGTCCAAG | 62 |  |
|  | 20 | CGCTGTTTCATGCTCCTC | CTTCCCCACTATGGGTCATC | 60 |  |
|  | 21 | CCTTCCCGCTGTTATCCAG | ACCTCATCCTGCCCTTCC | 56 |  |
|  | 22 | TAGACGCATGACCAGTGTTG | CAGCAAGCACAGCAATCAG | 60 |  |
|  | 23 | TCCTGCTGTGTGGTTAGACG | ACAGAAAACCCCTCTGAAAAG | 60 |  |
| ***ASXL1*** | 12 (1) | AGTCCCTAGGTCAGATCACCC | ATGGCTGGTCCCCAGTG | 60 | 3 |
|  | 12 (2) | GAGAGGACCTGCCTTCTCTG | ATTCTGGTTTGGGCTGTTTC | 56 |  |
|  | 12 (3) | CCTCCTATGAGGGAAAGTGATAC | GGACCGCACATACTCGAGAC | 56 |  |
|  | 12 (4) | CCCCACTGTCCAAGGTG | TCTTCACAACTAAAGGAAGAGAACTG | 56 |  |
|  | 12 (5) | AAAGAATTGCAAGGCAGTCC | CATCCCTTCCAAGTGACCC | 56 |  |
|  | 12 (6) | AGTTGGGACCAAGCACAAAC | TGACAGAGGGCTTTAGCAGTG | 60 |  |
| ***EZH2*** | 2 | GCACCTTTCTGTAAATGCCAAC | GACAGATCAAGAACCTAAGCT | 54 |  |
|  | 3 | GGATGGCAATCGTTTCCTG | CTACAGCAGTCATTAACAGTTGCAC | 54 |  |
|  | 4 | GGGAAGGGTTGGAGGTTTTG | ACTGCTTAACACTTTTCCTTTGGAG | 54 |  |
|  | 5 | TGCTTATTGGTGAGAGGGGTC | GCCCAGGTTCAGTCCCTTATAG | 54 |  |
|  | 6 | TTGCTTCCTTTGCCTAACACC | AGGATTACAGAGTTAGCCACCCTA | 54 |  |
|  | 7 | AGAGCTGGGAGTAGAACCTA | GCAGAGTACCACAAGTACACA | 54 |  |
|  | 8 | AGAGCCATTCCTTTATGTTTTAGGC | TGCAAAAGATTTCAGAGCAATCC | 54 |  |
|  | 9 | GAGCCTGAAGGAAAGTTGTATGAAG | TGGAAGAAACAGCATGGGTG | 54 |  |
|  | 10 | GTCTGACTCCGTGCACATTAGG | AGCAGGGCAAACACCACAA | 54 |  |
|  | 11 | GTGTTTTGCCGATTGGATTTGAG | GGGAAATTCTGTAACATACCTGGAGA | 54 |  |
|  | 12 | GGTAGGGACAGCGATGTGT | TCCCAGTGGAAATAAGATTCCATAG | 54 |  |
|  | 13 | CAGGAGTCATGGATGAACACAG | CTAAAGCACGGCTACATCTCAG | 54 |  |
|  | 14 | CGTTGTGGAAACCGGAATATGTG | TACAACTTTGCCTGCCTCAC | 54 |  |
|  | 15 | GGGTAATAAGGTGGTTGTGAGGG | AACTGAAGAGACTGCCCAAG | 54 |  |
|  | 16 | TGCCTTAGAACAAACAGGTCTGAG | AAGTCCAGGCTGAAAAGGCA | 54 |  |
|  | 17 | TACTGCGCAGACCGAATG | TTCCTTTCAAGCAAGCAGCC | 54 |  |
|  | 18-19 | GCTTTTGAGTCAGATAACCATCTTG | TGTAACCTAATTCCCCACTAATGC | 54 |  |
|  | 20 | TGAATGTGCCGTTATGCAGG | ACAAAACACTTTGCAGCTGGT | 54 |  |
| ***IDH1*** | 4 | CCAGTGCTAAAACTTGGCAG | AATTTCATACCTTGCTTAATGGG | 57 |  |
| ***IDH2*** | 4 | CTGTGTTGTTGCTTGGGGTT | GGGGTGAAGACCATTTTGAA | 55 |  |
| ***CBL*** | 8 | GGACCCAGACTAGATGCTTTCT | GAAAATACATTTCCTAGAGATCAAAA | 50 |  |
|  | 9 | CTGGCTTTTGGGGTTAGGTT | TCGTTAAGTGTTTTACGGCTTT | 50 |  |
| ***CBLB*** | 9 | TCAATTGTGAGATATTGAGTGTGC | AAAGCACTTACCAGCATTACTTCC | 50 |  |
|  | 10 | CCCTGTTCTATACCCACTACCAC | GCATCATTTCCTTTTCATATGGTC | 50 |  |
| ***CBLC*** | 7 | CCAAGTTGTGTCCCCGTG | TTTGGGGCTTTCCCTGTAG | 55 |  |
|  | 8 | AGGGACAGGATGGAGAGCAG | AGTCCCTCCCCACTCAGG | 60 | 3 |
